# Taking Neighborhood Health to Heart (TNH2H): Building a Community-Based Participatory Data System

**Published:** 2012-01-19

**Authors:** Deborah S. Main, George Ware, Patricia G. Iwasaki, Mark Burry, Emily Steiner, Katherine Fedde, Leah M. Haverhals

**Affiliations:** Department of Health and Behavioral Sciences, University of Colorado Denver. Dr Main is also on the TNH2H steering committee; Colorado Department of Public Health and Environment, Denver, Colorado. Mr Ware is also on the TNH2H steering committee; University of Denver, Denver, Colorado. Ms Iwasaki is also on the TNH2H steering committee; TNH2H steering committee; University of Colorado Denver, Denver, Colorado; University of Colorado Denver, Denver, Colorado; Denver Veteran's Affairs Medical Center, Denver, Colorado

## Abstract

**Background:**

*Healthy People 2020* calls for increased monitoring of local health and health disparities, but successful models of designing and implementing data collection systems for this purpose are lacking.

**Community Context:**

We describe the process, methods, and outcomes of a community-based participatory research initiative, Taking Neighborhood Health to Heart, designed to collect and disseminate comprehensive health data from 5 diverse urban neighborhoods in Denver, Colorado.

**Methods:**

Since its beginning in 2006, this initiative has involved community members in collection of individual health surveys from 1,146 households; audits of sidewalks, buildings, and other built environment features in 412 neighborhood blocks; audits of availability, price, and quality of fresh produce in 69 local stores; and audits of conditions and amenities in 20 local parks. Community members and researchers share, interpret, and disseminate these data through a joint data review and dissemination committee.

**Outcome:**

Through our community-based data collection system, Taking Neighborhood Health to Heart has been able to collect, analyze, and disseminate locally relevant data on people and neighborhoods to monitor heath and health disparities.

**Interpretation:**

Since 2006, the initiative has sustained its focus on community-based participatory research that targets collection and dissemination of local health data. We have used this information to identify salient health issues and advocate for neighborhood programs, policies, and environmental changes to built and social features of neighborhoods that have historically led to unequal opportunities and social disadvantage.

## Background

With the release of *Healthy People 2020* and the Institute of Medicine's recent report calling for more effective community-based approaches to measure public health improvements, there is growing national interest in better data systems for understanding and monitoring health and health disparities at the local level (1,2). Although statewide systems such as the Behavioral Risk Factor Surveillance System (BRFSS) are invaluable for monitoring health at state and county levels, they are not designed to provide health data for smaller geographic areas like neighborhoods (3,4). The national call for addressing the *Healthy People 2020* objectives at the local level necessitates dramatic shifts in how we plan, implement, and sustain health information systems for monitoring progress toward these goals. This shift also includes a focus on collecting a broader range of local health indicators that reflect social determinants of health, yet we have few examples in the literature of strong models of local data collection (5-7).

## Community Context

One such example is a community-based participatory research (CBPR) initiative called Taking Neighborhood Health to Heart (TNH2H). Initially funded by the National Institutes of Health, TNH2H involves 5 contiguous neighborhoods in the Denver metropolitan area located in and around the former Stapleton Airport site. Stapleton, redeveloped as a mixed-use, active-living neighborhood, is adjacent to 4 neighborhoods that endured more than 60 years of airport noise, traffic, and pollution — Northeast Park Hill, Park Hill, East Montclair, and Northwest Aurora — each with its own historical, political, economic, and cultural identities. The 5 TNH2H neighborhoods are racially, ethnically, and economically diverse (Table 1).

TNH2H began in June 2006 with the primary objective of developing a new community-academic partnership and collecting and disseminating local data on the health of people and neighborhoods. This objective proved synergistic with the research aim of studying the effect of built and social environments on health and health disparities. With help from the Stapleton Foundation, a local nonprofit organization, university researchers reached out to community members in the 5 neighborhoods. The first TNH2H Council convened in January 2007 and throughout the past 5 years has involved more than 100 community residents and local leaders from each neighborhood. Its members reflect the racial, ethnic, and economic diversity of the study area. The TNH2H Council and subcommittees (ie, steering committee, food committee, data review and dissemination committee) meet at least monthly.

The purpose of this case study is to describe the process, methods, and outcomes of TNH2H. The goal of this initiative is to build strong partnerships with neighborhood-based organizations, schools, and other groups to use the data to improve health and decrease health disparities.

## Methods

In line with TNH2H objectives to collect a broad set of health measures, we used 1) qualitative focus groups, 2) random household health surveys, 3) audits of the built environment, 4) audits of neighborhood parks and grocery stores, and 5) strong participatory processes to involve the community in the design and implementation of data collection, interpretation, and dissemination of findings. This research was approved by the Colorado Multiple Institutional Review Board (COMIRB no. 06-0624).

### Focus groups

From October through November 2006, we conducted focus groups to understand the health issues, priorities, and resources within and among the 5 neighborhoods as well as community perceptions of access and barriers to healthy foods, local parks and recreation centers, and medical services. We used snowball sampling to convene 5 focus groups, 1 in each TNH2H neighborhood, consisting of 5 to 11 community residents, for a total of 36 participants. Experienced facilitators conducted the groups, digitally recording and transcribing all sessions. We ended each session by asking participants to join the TNH2H Council. We invited them to attend (and bring fellow residents to) a community meeting where we shared focus-group findings, facilitated a discussion about neighborhood health, and described the principles of CBPR and the TNH2H project.

### Recruitment and survey

From January through April 2007, the TNH2H Council used focus group findings and other community input to determine the content of a random household survey. The household survey included BRFSS and other well-validated health measures for a broad set of social determinants of health (eg, perceived safety and social cohesion of neighborhoods, racial/ethnic discrimination, food insecurity, and stress). We used TNH2H Council meetings to pilot the survey and review the protocol. The final survey was available in English and Spanish.

Although we originally planned a random-digit-dialed (RDD) telephone survey, the TNH2H Council was concerned about missing participants who did not have access to a telephone, only used cellular telephones, or were reluctant to participate in RDD "cold calls" — a concern supported by some studies (5,6). On the basis of community recommendations, we designed a 2-phase method: 1) training community residents to recruit participants at the door for the telephone survey, followed by 2) computer-assisted telephone interviews (CATI) conducted by a local agency.

From May through December 2007, trained community recruiters used detailed maps of randomly selected blocks (based on a sampling scheme that adjusted for percentage of households in each neighborhood and stratified on the basis of block population density), household starting points, and walking direction within blocks to recruit participants. Recruiters worked in pairs for safety (at least 1 was bilingual) and wore TNH2H shirts and photo identification badges. One week before recruiting, flyers were placed on doors within randomly selected blocks to notify residents about the project. When someone in the household answered the door, recruiters determined eligibility, selected the index adult using the "birthday method" (adult with the next birthday), and then asked 18 preliminary questions about the household (which cut down the survey length). The recruiters completed appointment cards for the index adult by listing the best times for the CATI unit to call. If the adult agreed to participate, his or her contact information and preferred calling times were shared with the CATI unit. When no one was home, recruiters left a door hanger with an invitation to participate in the survey. From June 2007 through February 2008, the CATI unit conducted the household survey in English and Spanish. The survey took an average of 20 to 25 minutes; all participants were mailed a $10 gift certificate after completing the survey.

### Audits of the built environment

From June through December 2007, we conducted neighborhood audits of the built environment (simultaneous to conducting household surveys). The audit included structural features that promoted or hindered walking and biking in the neighborhood; aesthetic features that reflected social disorganization, safety, and crime; and the overall condition of houses and buildings. We used established, psychometrically tested measures (10-12) but also relied on community input to include measures salient to residents. The final audit tool included 58 questions. Following community recommendations, researchers hired and trained 4 pairs of graduate students (not from neighborhoods) to conduct the built-environment audits within blocks surrounding surveyed households to link survey data with built environment data.

### Audits of local parks, grocery stores

From October through December 2007, 1 pair of trained auditors conducted detailed assessments of all 20 parks in the 5 neighborhoods using the BRAT-Direct Observation, a reliable and valid tool designed to measure the environmental determinants of physical activity in parks (13,14). Trained auditors used the tool to assess features, condition, access, aesthetics, safety, rules, and activity areas (courts, sports fields, pools, playgrounds) of each park. Parking availability and safety were assessed, and streets that border or cross the park were also measured for safety, access, and design.

In response to community interest, we audited all local grocery stores from June through October 2009 to supplement neighborhood built-environment data on blocks and parks. Using an adapted version of the US Department of Agriculture (USDA) Community Food Security Assessment Toolkit (15), we collected data on the availability, price, and quality of 97 food items in 69 local food stores and retailers in the 5 neighborhoods.

### Community involvement

Among the first steps of the community-academic partnership was to develop operating agreements (posted at every TNH2H Council meeting), which included valuing the perspectives of all members, establishing a process for airing and resolving disputes, and meaningfully involving community members (TNH2H Council and subcommittees) in all phases of data collection, analysis, interpretation, and dissemination. "Meaningful involvement" included translating meeting materials into Spanish and having a Spanish interpreter present at meetings.

Before completing data collection, the TNH2H Data Review and Dissemination (DRAD) committee was formed, composed of both researchers and community members. The goal of the DRAD was to develop standards and processes for managing and sharing data both internally and among a variety of academic and community groups. Among the DRAD's first priorities was to educate the larger TNH2H Council about the historical research abuses within poor, racial/ethnic minority communities, reinforcing the importance of community input, involvement, and decision making in how local health data are collected, analyzed, interpreted, and disseminated. After the data were collected, DRAD developed a written data request form to be completed by all community and academic groups interested in using TNH2H data. This form included questions about intended uses of the data and how the community was to be involved in and benefit from the request. The second priority of DRAD was to share the health data with each of the 5 neighborhoods, through community presentations in neighborhood meetings and publications in neighborhood newspapers and newsletters.

An early community concern in the data dissemination process was that some neighborhoods might be stigmatized by how the data were presented, for example, when perceptions of neighborhood safety differed significantly for 2 adjacent neighborhoods. DRAD decided that presentations of findings would not compare neighborhoods unless data were deidentified. These sensitivities remain in place today as the data are more widely requested and used.

## Outcome

As a result of the CBPR data collection methods and processes, TNH2H has demonstrated important outcomes and lessons learned about the challenges and opportunities of local efforts.

### Effective use of focus groups early in the process

Approximately 1 month after we conducted focus groups, we held a community meeting, "Community Conversations about Health," that brought together people from all 5 neighborhoods. Twenty community members from the 5 neighborhoods and 9 staff members attended this meeting, which introduced CBPR, invited community members to become involved in the TNH2H Council, and shared summary findings of focus groups. These findings were used in subsequent TNH2H Council meetings to identify neighborhood health priorities and relevant content for local data collection efforts, including the household survey (Table 2).

### Success in reaching diverse populations through household surveys

We used the 2-stage data collection process (with community recruiters) to reach more diverse populations (similar to the composition of neighborhoods) than reflected in state and even county health data. Compared to 2000 census data, the TNH2H survey sample was similar in racial/ethnic composition — an important finding for enhancing the credibility of our approach among the diverse neighborhoods. There were, however, differences in our TNH2H sample compared to the census. In 2 neighborhoods, the survey sample underrepresented households making less than $25,000 and, in 1 neighborhood, overrepresented households making less than $25,000. The survey sample also underrepresented adults with less than a high school education. The Denver County BRFSS sample is different from the TNH2H survey sample in terms of race/ethnicity and income — not surprising, because BRFSS data are intended to represent Denver County and not individual neighborhoods (Table 1). However, these demographic differences illustrate how county-level surveillance data may not necessarily reflect common and unique issues related to health and health disparities for their different neighborhood populations.

Throughout the recruitment process, the community recruiters left flyers and/or interacted with more than 6,000 households to recruit the study sample. One of the difficulties recruiters experienced was gaining access to locked apartment buildings. Although our sampling strategy included a high percentage of blocks with renters (57% of sampled blocks), the percentage of renters recruited by community members in our sample was 36%; at least some of this difference was attributable to not getting into locked apartment buildings and potentially oversampling residents who rent homes or townhomes.

Community members recruited 57% of eligible adults to take the telephone survey (range, 53%-66% among the 5 neighborhoods). Overall, 64% of residents recruited by community members at the door participated in the telephone survey. Of people recruited, the CATI unit averaged 11 unsuccessful call attempts until survey completion. The average time between successful recruitment and completion of a survey was 38 days (median, 25 days). When we noticed an unusually long "lag" between time of recruitment and completed telephone interviews, 2 community recruiters called both English- and Spanish-speaking adults to verify their continued willingness to participate.

After combining at-the-door recruitment rates with completion rates, the final response rate was 36% of eligible adults completing the CATI survey (range, 31%-45% among the 5 neighborhoods).

The litmus test was whether local findings were able to reflect meaningful differences and similarities in health and health disparities among the 5 neighborhoods (ie, whether they had "face validity" for the community). Many of our findings on health and social determinants varied drastically among neighborhoods (Table 3); thus, efforts to understand and improve health must also consider broader forces like the safety of neighborhoods, trust in one's neighbors, and how factors like discrimination and the built environment affect health in complex ways.

### Analysis and use of neighborhood audit data

We collected comprehensive audit data on neighborhood blocks, parks, and grocery stores, which are now receiving focused attention. For example, park and neighborhood audit data are being used for a dissertation research project on childhood obesity that links childhood obesity data to data on the built environment and distance to and features of local parks. Because of strong community interest in food, we prioritized analysis of grocery store data and found variability in local stores that sell fresh fruits and vegetables: 24 of the stores did not sell any of the 22 fruits and vegetables on our list. Additionally, the price of a fruit and vegetable basket, made up of 5 standard fruits and vegetables, ranged from $4.11 to $16.82 among stores. A primary dissemination outcome of our grocery store audit in 2010 was the development of detailed, easy-to-read maps and a brochure in English and Spanish that clearly identifies availability and price of healthy food in 69 local grocery stores. The food audits also spurred an interest among some TNH2H Council members to create a subcommittee to focus on increasing food access in the 5 neighborhoods.

### Community involvement in data collection, analysis, interpretation, and dissemination

Warnings from academic and community colleagues that "community members do not care about data" were unfounded. We have involved more than 100 residents and community leaders in this CBPR data collection and dissemination initiative. This engagement continues today despite a lack of stable funding. TNH2H's history of involving community members in all phases of data collection and dissemination has been among its greatest successes. For each neighborhood, we developed a set of health briefs on the primary survey topics in English and Spanish, summarizing its results compared to the combined neighborhoods' results. Community members and researchers have presented TNH2H processes and findings at local, state, and national meetings. The DRAD committee has successfully competed for funding from University of Colorado Denver's Clinical Translational Science Award to expand data collection to adults in locked apartment buildings and to use "house meetings" (meetings of 6-10 neighborhood residents hosted in community members' homes) as an innovative data collection and dissemination strategy for reaching new populations within the diverse neighborhoods.

## Interpretation

After 5 years of developing and implementing processes and methods for our CBPR data system, we have created a useful model and honed principles based on our experiences, learning, and successes. First, we believe that a CBPR model is essential for enhancing the relevance, quality, value, and use of local data. This model requires that community members are engaged in all phases of the data collection, interpretation, and dissemination process — and that community and researchers share data and make decisions together. Second, if we are to realize improvements in the health of people and communities in line with *Healthy People 2020*, the level of measurement and broader focus on social determinants of health are essential; quantitative data help the community understand and track its health and health priorities, and qualitative data reflect more in-depth understanding and narrative of the experience of community members and their solutions for promoting health. Finally, to be most effective, a CBPR data system should include strong mechanisms for updating and supplementing data to monitor and address health gaps and for disseminating information in useful formats (eg, low-literacy handouts in multiple languages, easy-to-read maps of available resources, house meetings for sharing and story telling). Together, these principles help ensure that information remains locally relevant and spurs action and community change. TNH2H has achieved this relevance by, for example, sharing data for 2 local health impact assessments, 1 currently targeting improvements in a neighborhood recreation center. That TNH2H data are local and credible makes these and other uses possible; however, sustaining capacity to continue collecting, analyzing, and disseminating local health data over time is challenging; without substantial, stable funding, initiatives like ours would be difficult to maintain.

## Figures and Tables

**Table 1.** Demographic Characteristics of Neighborhoods, by TNH2H Survey, US Census, and BRFSS, Denver, Colorado, 2007

Neighborhood/Survey	n (SD)^a^	Race/Ethnicity, n (%)		Income <$25,000, n (%)	<High School Education, n (%)

		African American	Hispanic/Latino		
**East Montclair (Denver)**					
Census 2000	7,691 (4,218)	2,461 (32)	2,462 (32)	3,153 (41)	2,153 (28)
TNH2H survey sample	146	34 (23)	38 (26)	46 (32)	21 (14)
**Northeast Park Hill (Denver)**					
Census 2000	8,794 (2,744)	6,068 (69)	2,111 (24)	3,605 (41)	2,989 (34)
TNH2H survey sample	95	65 (68)	21 (22)	60 (63)	21 (22)
**Park Hill (Denver)**					
Census 2000	19,202 (7,522)	6,913 (36)	1,920 (10)	4,032 (21)	2,304 (12)
TNH2H survey sample	258	73 (28)	23 (9)	33 (13)	6 (2)
**Northwest Aurora^b^**					
Census 2000	24,399 (7,773)	3,659 (15)	14,151 (58)	2,516 (10)	11,712 (48)
TNH2H survey sample	237	35 (15)	131 (55)	74 (31)	89 (38)
**Stapleton (Denver)^c^**					
Population estimate	6,446 (1,871)	NA	NA	NA	NA
TNH2H survey sample	214	6 (3)	16 (7)	6 (3)	0
**5 neighborhoods combined**					
Census 2000 (excluding Stapleton)	66,532 (24,128)	19,101 (29)	20,644 (31)	13,306 (20)	19,158 (29)
TNH2H survey sample	950	229 (24)	213 (22)	208 (22)	137 (14)
Denver County BRFSS 2007-2008 sample	1,675	134 (8)	436 (26)	452 (27)	251 (15)

Abbreviations: TNH2H, Taking Neighborhood Health to Heart; BRFSS, Behavioral Risk Factor Surveillance System; SD, standard deviation; NA, not available.

a Only Census 2000 values and the population estimate for Stapleton (Denver) include SDs.

b Northwest Aurora is not in Denver County.

c No 2000 Census data available for Stapleton (new development); population estimate for Stapleton from the Piton Foundation (http://www.piton.org/).

**Table 2.** Results of TNH2H Focus Group to Identify Community Health Issues and Priorities, Denver, Colorado, 2007

**Question/Issue**	**Community Responses**
What you like about your neighborhood	Community pride; traditions, new and old; know your neighbors; longtime residents; old established neighborhoods; community connections; location ("we are close to everything!")
What health issues you worry about	Safety (crime, traffic); lack of access to health care; chronic illness: diabetes, high blood pressure, high cholesterol, arthritis; overweight/obesity; unhealthy behaviors: smoking, lack of exercise, poor eating habits; environmental hazards: pollution, toxins
Physical activity and access to healthy food in your neighborhood	Access to places to be physically active differs from neighborhood to neighborhood; access to healthy food differs from neighborhood to neighborhood
Barriers to being physically active and eating healthy food	Poor public transportation; no or poor sidewalks; unsafe streets; cost, inconvenience; lack of time; no healthy restaurants, grocery stores; rarely see people being active; lack of places for kids to go; lack of walking or bike paths
What would help	Better and more accessible programs in the community (eg, schools, churches, health care); safe streets; safe recreation centers and parks; better access to low-cost healthy food; improved communication about what's available for exercise and healthy eating
Impact of new development and revitalization (Stapleton)	Generally view changes as positive; greater access to shops, parks, walking/biking paths for some; some worry about impact on own neighborhoods, want to maintain neighborhood identity; changes will affect some neighborhoods more than others
Next steps	Increase ongoing community participation in this project ("starting tonight!"); talk with more people in communities ("who else should we talk to? who are we missing?"); design survey of households and neighborhoods

Abbreviation: TNH2H, Taking Neighborhood Health to Heart.

**Table 3.** Health, Social, and Neighborhood Characteristics, TNH2H Household Survey, Denver, Colorado, 2007

Measure	Neighborhood^a^						State, %^b^
	N1	N2	N3	N4	N5	All	
**Health**							
Eat "5 A Day" fruits and vegetables^c^, %	21	12	20	20	17	19	NA
Meet physical activity recommendations^d^, %	46	41	33	65	66	52	55
Ran out of food money in past year, %	30	46	41	11	3	23	NA
Delayed health care because of cost, %	38	41	46	21	12	30	NA
Overweight (BMI, 25.9-30.0 kg/m^2^), %	26	42	33	33	29	32	36
Obese (BMI, ≥30.0 kg/m^2^), %	25	37	33	19	9	22	19
Current smoker, %	30	36	17	17	8	19	19
**Social and neighborhood**							
Agree that people in neighborhood can be trusted, %	60	50	41	81	90	67	NA
Agree that this is a close-knit neighborhood, %	49	64	41	70	85	62	NA
Agree that the crime in neighborhood makes it unsafe to walk at night, %	56	51	62	32	85	40	NA

Abbreviations: TNH2H, Taking Neighborhood Health to Heart; NA, not available; BMI, body mass index.

a Neighborhood data were deidentified to avoid stigmatizing neighborhoods.

b Source: 2007 Colorado Behavioral Risk Factor Surveillance System.

c Source: http://www.fruitsandveggiesmatter.gov/.

d Physical activity was calculated using the International Physical Activity Questionnaire (16). Meeting physical activity recommendations requires at least 30 minutes of moderate or vigorous physical activity daily.
